# Three-time-point directional patterns of frailty and physical function in older adults participating in community gathering places in Koshigaya City, Japan: a retrospective exploratory study

**DOI:** 10.1016/j.pmedr.2026.103646

**Published:** 2026-09-21

**Authors:** Keisuke Kubota, Takaya Abe, Takashi Nasu, Yu Shimano, Hiroo Furusawa, Toyohiro Hamaguchi, Yoshinori Kitabatake, Yayoi Amakusa

**Affiliations:** a Research and Development Center, Saitama Prefectural University, Saitama 343-8540, Japan; b Rehabilitation Amakusa Hospital, Saitama 343-0002, Japan; c Koshigaya-Seiwa Hospital, Saitama 343-0856, Japan; d Saiyu Clinic, Saitama 343-0823, Japan; e School of Health and Social Services, Saitama Prefectural University, Saitama 343-8540, Japan

**Keywords:** Frailty, Kihon checklist, *Kayoinoba*, Physical function, Longitudinal trajectories, Older adults

## Abstract

**Objective:**

Community gathering places (Kayoinoba) promote social participation and may help prevent frailty among older adults. This exploratory study examined three-time-point trajectories of frailty and physical function and whether baseline multidimensional information helped distinguish favorable long-term trajectories.

**Methods:**

This retrospective longitudinal study used municipal assessment data from Kayoinoba sites in Koshigaya City, Japan, collected at baseline (September 2016–October 2019), approximately 6 months later (March 2017–October 2020), and at the fiscal year 2022 municipal evaluation, conducted between August 2022 and November 2023. Outcome-specific samples included Kihon Checklist (KCL) total score (*n* = 95), Timed Up and Go (TUG) test (*n* = 74), one-leg standing time (OLS; *n* = 103), and 30 s Chair-Stand Test (CS-30; *n* = 100). Participants were classified into five prespecified direction-based trajectory patterns.

**Results:**

Outcomes showed heterogeneous longitudinal patterns. CS-30 had the highest LongGood (51.00%) and ImproveMaintain (38.00%) rates. TUG had the lowest LongGood rate (27.03%) and frequent ImproveRebound (37.84%) and ConsistentDecline (28.38%) patterns. Domain-score models showed numerically higher apparent areas under the receiver operating characteristic curve for several outcomes, but optimism-corrected estimates were attenuated.

**Conclusions:**

Frailty and physical function showed heterogeneous, outcome-dependent directional patterns. Findings are descriptive and hypothesis-generating, not causal evidence of Kayoinoba effectiveness.

## Highlights

• Frailty trajectories varied substantially among community-dwelling older adults.
• Three-time-point assessments identified distinct frailty outcome patterns.
• Physical function measures showed heterogeneous long-term trajectories.
• Baseline multidimensional frailty profiles differed across trajectory groups.
• Findings support targeted community-based frailty prevention strategies.

## 1 Introduction

Prevention of frailty in rapidly aging societies requires scalable, community-based strategies that preserve functional independence and social participation. In Japan, the Kihon Checklist (KCL) is a valid and widely used multidimensional screening tool for assessing frailty, predicting future disability, and identifying individuals who would benefit from long-term care (Fukutomi et al., 2015; Satake et al., 2016; Sewo Sampaio et al., 2016; Watanabe et al., 2022). Longitudinal studies have also demonstrated its utility for monitoring frailty transitions, incidence, and recovery, highlighting its value as a dynamic assessment tool (Hirose et al., 2022; Imai et al., 2025; Imai et al., 2023; Ohashi et al., 2021; Yang et al., 2024).

Social participation is an important modifiable determinant of frailty trajectories in older adults. Previous studies have shown that social engagement and community-level social capital are associated with lower frailty incidence, reduced disability and mortality risk, and more favorable transitions, even among frail individuals (Abe et al., 2023; Takeuchi et al., 2023; Ihara et al., 2022; Katayama et al., 2022; Noguchi et al., 2022; Saeki et al., 2023; Takatori and Matsumoto, 2023). Within this context, community gathering places (*Kayoinoba*) have been widely promoted in Japan. These resident-centered settings combine structured exercise with social interaction and mutual support. Studies from multiple municipalities suggest that continuous participation may suppress functional decline, improve lower-extremity strength, and delay progression from robustness to frailty (Hayashi et al., 2021a, Hayashi et al., 2021b; Mori et al., 2025; Tajika et al., 2022; Tsuji et al., 2024; Uemura et al., 2025).

In Koshigaya City, studies of an exercise-focused Kayoinoba program have shown that six months of participation is associated with improvements in caregiving risk and motor function (Abe et al., 2024). Furthermore, baseline mobility measures such as the Timed Up and Go (TUG) test predict short-term changes in KCL scores (Nasu et al., 2025), while age-specific analyses indicate that long-term participants maintain relatively favorable functional profiles despite age-related decline (Kubota et al., 2025).

Despite these findings, an important gap remains. Most studies of community-based programs have focused on average short-term changes and have not examined long-term heterogeneity in outcomes. Longitudinal studies of physical performance suggest that decline is rarely uniform; individuals may follow distinct trajectories, including maintenance, gradual decline, or deterioration, across different functional domains (Hoekstra et al., 2020; Tange et al., 2022; Taniguchi et al., 2016). Whether Kayoinoba participants follow similar or diverse long-term trajectories remains unclear.

Therefore, this exploratory study aimed to examine three time-point trajectories of frailty and physical function among older adults participating in community gathering places in Koshigaya City. We hypothesized that trajectories would be heterogeneous rather than uniform and assessed whether baseline KCL domain-level information provided additional discrimination for favorable long-term trajectories compared with the KCL total score alone. Sensitivity analyses were also used to examine the robustness of the findings to age eligibility and variation in long-term follow-up duration.

## 2 Methods

### 2.1 Study design and population

This retrospective longitudinal study included an exploratory discrimination analysis. Kayoinoba is a municipality-supported program conducted at 46 community sites in Koshigaya City. Participation was open to all interested residents without specific eligibility criteria. Most sites met once or twice weekly for approximately 90 min to 2 h per session. Trained resident leaders delivered a common structured exercise program, while social and recreational activities varied by site. The exercise component lasted approximately 40–60 min and consisted of four core elements: dynamic stretching as a warm-up; resistance exercises for the upper and lower limbs using elastic bands (“Koshigaya Rakunobi Exercise”); exercises performed while singing (“Rakusho Exercise”); and static stretching as a cool-down. Physical therapists and municipal staff trained the resident leaders and provided exercise instruction and periodic on-site guidance. These four core exercise components were consistently implemented across sites, although session frequency and other social or recreational activities varied (Abe et al., 2024). Because no non-participant comparison group was available, Kayoinoba participation was treated as the observational setting rather than an exposure for causal inference.

The source dataset included 1132 participants assessed within the municipal Kayoinoba program. Baseline, 6-month, and fiscal year 2022 (FY2022) data were linked using individual identification numbers. At least one value was available for 763 participants at baseline, 595 at 6 months, and 604 in FY2022; 204 had at least one value in all three assessment periods.

Because data availability differed across outcomes, outcome-specific complete-case samples were created. Participants were included when numeric values were available at all three time points for each outcome, whereas non-numeric or unavailable entries were treated as missing. Analytic sample sizes were 95 for KCL total score, 74 for TUG, 103 for OLS, and 100 for CS-30. Because Kayoinoba had no age-based eligibility criterion, the municipal dataset included a small number of participants younger than 65 years. Participants younger than 65 years represented 6.32%, 4.05%, 6.80%, and 6.00% of these samples, respectively; the overall age range was 56–85 years. Sensitivity analyses therefore repeated trajectory analyses among participants aged ≥65 years. Because outcome-specific samples differed, direct comparisons between samples were not performed.

Assessment dates were available for the baseline, 6-month, and FY2022 evaluations. Baseline assessments were conducted between September 2016 and October 2019, approximately 6-month assessments between March 2017 and October 2020, and the FY2022 municipal evaluation was conducted between August 2022 and November 2023. Participant-specific follow-up intervals were calculated directly from the recorded assessment dates. Follow-up durations were summarized using the median, interquartile range, and range for each outcome-specific analytic sample.

### 2.2 Measures

Outcomes were KCL total score, TUG (Podsiadlo and Richardson, 1991), CS-30 (Jones et al., 1999), and OLS (Michikawa et al., 2009). Physical performance was assessed at each site by physical or occupational therapists using the same protocol across assessments; assessors practiced the procedures beforehand. TUG was performed once over 3 m using a standard folding chair without assistive devices. OLS was measured once per side with a 120-s ceiling, and bilateral values were averaged. CS-30 was performed once using the same type of chair, recording complete repetitions within 30 s. These measurements were obtained as part of routine municipal assessments and were extracted from the existing municipal dataset for the present retrospective analysis.

Baseline candidate predictors included age, sex, KCL total score, seven KCL domain scores (activities of daily living, physical function, nutrition, oral function, outdoor activities, cognitive function, and depressive mood), baseline TUG, OLS, CS-30, and Life-Space Assessment total score (Ullrich et al., 2022).

### 2.3 Statistical analysis

To characterize longitudinal heterogeneity, participants were classified into five prespecified trajectory patterns based on changes between baseline, 6 months, and the FY2022 evaluation. Because lower values indicate better status for KCL and TUG, whereas higher values indicate better performance for OLS and CS-30, all outcomes were transformed to a favorable-direction scale in which higher values indicate better status. KCL and TUG were multiplied by −1, whereas OLS and CS-30 retained their original direction.

Let Δ01, Δ12, and Δ02 denote changes from baseline to 6 months, from 6 months to FY2022, and from baseline to FY2022, respectively. Positive values indicated improvement, negative values indicated worsening, and zero indicated no change. The five mutually exclusive patterns were defined as follows: ImproveMaintain, Δ01 > 0 and Δ02 ≥ 0; ImproveRebound, Δ01 > 0 and Δ02 < 0; ConsistentDecline, Δ01 < 0 and Δ12 < 0; DelayedImprove, Δ01 ≤ 0 and Δ02 > 0; and StableOrOther, all remaining patterns.

These categories were based on prespecified direction-based rules rather than latent class growth modeling and should be interpreted as descriptive patterns. Because validated minimal clinically important differences were unavailable for all outcomes, the primary classification was based on direction of change. A sensitivity analysis treated changes smaller than 0.20 × the baseline standard deviation (SD) as no meaningful change. LongGood was defined separately as maintenance of, or improvement beyond, baseline status at the FY2022 evaluation (Δ02 ≥ 0 on the favorable-direction scale).

Baseline characteristics were summarized as mean ± SD or n (%). Longitudinal changes were examined using Friedman tests and linear mixed-effects models on the original outcome scales. Time was modeled categorically (baseline, 6 months, FY2022), with participant-specific random intercepts, common residual variance conditional on the random effect, maximum-likelihood estimation, and Satterthwaite degrees of freedom. Assumptions were assessed using standardized conditional residual-versus-fitted plots and Q–Q plots of residuals and random intercepts and were considered satisfactory.

To account for variability in long-term follow-up duration, a sensitivity linear mixed-effects model additionally included each participant's actual baseline-to-FY2022 follow-up duration, calculated from the recorded assessment dates, as a fixed effect for the FY2022 observation. Pairwise changes were summarized using within-person mean differences, standardized effect sizes, and 95% confidence intervals. *P* values were interpreted descriptively without multiplicity adjustment.

Trajectory distributions were summarized for each outcome. LongGood and ImproveMaintain were used as binary targets for exploratory discrimination analyses. The TotalModel included age, sex, baseline TUG, OLS, CS-30, Life-Space Assessment, and KCL total score; the DomainModel replaced KCL total score with its seven domain scores. Complete-case logistic regression models were evaluated using the area under the receiver operating characteristic curve with bootstrap 95% confidence intervals and optimism correction. Because of small event numbers and use of the same data for model development and evaluation, estimates were considered exploratory rather than externally validated.

The study was conducted in accordance with the Declaration of Helsinki and was approved by the Ethics Committee of Saitama Prefectural University (approval no. 25101). Written informed consent was obtained from all participants, and data were handled in accordance with institutional requirements for the protection, safety, and privacy of human participants. All statistical analyses were performed using MATLAB R2024a (MathWorks, Natick, MA, USA).

## 3 Results

### 3.1 Participant flow and longitudinal changes

The source dataset included 1132 participants; 763 had baseline data, 595 had 6-month data, and 604 had FY2022 data, with 204 having at least one value in all three periods. Outcome-specific complete-case samples were KCL (*n* = 95), TUG (*n* = 74), OLS (*n* = 103), and CS-30 (*n* = 100) (Fig. 1). (See Table 1.)

**Fig. 1:**
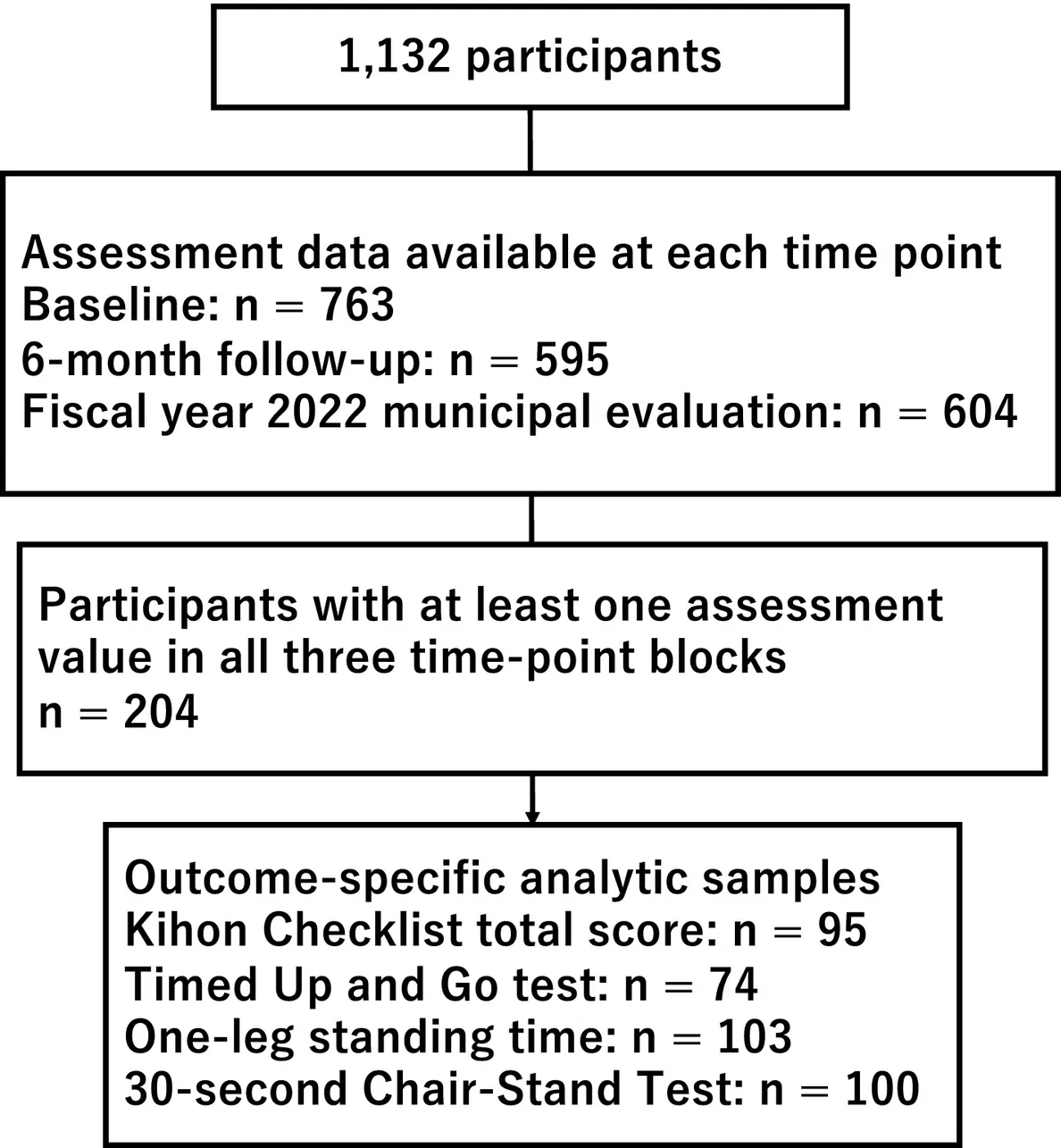
Flow of adults participating in community gathering places in Koshigaya City, Japan, from baseline through the fiscal year 2022 municipal evaluation, conducted between August 2022 and November 2023, and selection of outcome-specific analytic samples. The source dataset included 1132 participants. Data across assessment periods were linked using individual identification numbers. Baseline assessments were conducted between September 2016 and October 2019, approximately 6-month assessments between March 2017 and October 2020, and long-term municipal assessments between August 2022 and November 2023. Numbers at each assessment period indicate participants with at least one recorded assessment value. Because data availability differed across outcomes, outcome-specific analytic samples were created for the Kihon Checklist total score, Timed Up and Go test, one-leg standing time, and 30 s Chair-Stand Test.

**Table 1:** Baseline characteristics of adults participating in community gathering places in Koshigaya City, Japan, at baseline assessments conducted between September 2016 and October 2019.

	Kihon checklist (*n* = 95)	Timed Up and Go (*n* = 74)	One-leg standing (*n* = 103)	30 s Chair-stand (*n* = 100)
Age, years	74.28 ± 5.85	74.91 ± 5.39	74.30 ± 5.77	74.35 ± 5.71
Female, n (%)	86 (90.53)	66 (89.19)	94 (91.26)	91 (91.00)
Kihon Checklist, total score	3.43 ± 2.70	3.53 ± 2.83	3.41 ± 2.70	3.22 ± 2.57
Timed Up and Go test, s	6.54 ± 1.31	6.57 ± 1.38	6.51 ± 1.29	6.48 ± 1.23
One-leg standing time, s	40.72 ± 38.93	42.99 ± 39.87	40.25 ± 37.89	40.73 ± 38.21
30 s Chair-Stand Test, repetitions	21.00 ± 5.50	20.68 ± 5.74	21.15 ± 5.30	21.14 ± 5.27
Life-Space Assessment total score	82.68 ± 20.24	83.36 ± 20.58	82.08 ± 19.96	83.53 ± 20.04

Data are presented as mean ± standard deviation (SD) unless otherwise indicated. Because outcome-specific complete-case samples were used, the analytic samples were not identical across outcomes. No formal statistical comparisons were conducted among the outcome-specific samples.

Median baseline-to-FY2022 follow-up duration calculated from the recorded assessment dates was 4.79 years for KCL (interquartile range [IQR], 4.12–4.94; range, 3.72–5.17), 4.14 years for TUG (IQR, 3.79–4.94; range, 3.72–5.17), 4.79 years for OLS (IQR, 4.11–4.94; range, 3.72–5.17), and 4.79 years for CS-30 (IQR, 4.08–4.94; range, 3.72–5.17). In sensitivity analyses accounting for actual follow-up duration, the time effect remained evident for all outcomes, and adjustment did not materially alter the overall interpretation of longitudinal change.

KCL and TUG worsened by FY2022, OLS declined, and CS-30 initially improved before declining (Table 2; Fig. 2). Friedman tests and mixed-effects models indicated time-related changes for all outcomes. Baseline-to-FY2022 changes were 1.33 KCL points (95% CI, 0.82, 1.83), 1.18 s for TUG (95% CI, 0.59, 1.76), −11.15 s for OLS (95% CI, −16.54, −5.76), and −0.78 repetitions for CS-30 (95% CI, −2.31, 0.75).

**Table 2:** Longitudinal changes in frailty and physical function among adults participating in community gathering places in Koshigaya City, Japan, from baseline through the fiscal year 2022 municipal evaluation, conducted between August 2022 and November 2023.

Outcome	n	Baseline, mean ± SD	6 months, mean ± SD	Fiscal year 2022 evaluation, mean ± SD	Baseline-to-Fiscal Year 2022 change, mean (95% Confidence Interval)	Standardized paired effect size
Kihon Checklist total score	95	3.43 ± 2.70	3.47 ± 2.57	4.76 ± 3.19	1.33 (0.82, 1.83)	0.54
Timed Up and Go test, s	74	6.57 ± 1.38	6.54 ± 1.51	7.75 ± 3.32	1.18 (0.59, 1.76)	0.47
One-leg standing time, s	103	40.25 ± 37.89	39.94 ± 39.21	29.10 ± 22.25	−11.15 (−16.54, −5.76)	−0.40
30 s Chair-Stand Test, repetitions	100	21.14 ± 5.27	22.97 ± 6.36	20.36 ± 8.02	−0.78 (−2.31, 0.75)	−0.10

Data are presented as mean ± standard deviation (SD). KCL and TUG are lower-is-better outcomes, whereas OLS and CS-30 are higher-is-better outcomes. Baseline-to-FY2022 change represents the raw within-person change from baseline to the FY2022 evaluation. Positive values indicate increases in the original scale. For KCL and TUG, higher values indicate worse status; for OLS and CS-30, lower values indicate worse performance. The standardized paired effect size was calculated as the mean baseline-to-FY2022 change divided by the standard deviation of the within-person change scores.

**Fig. 2:**
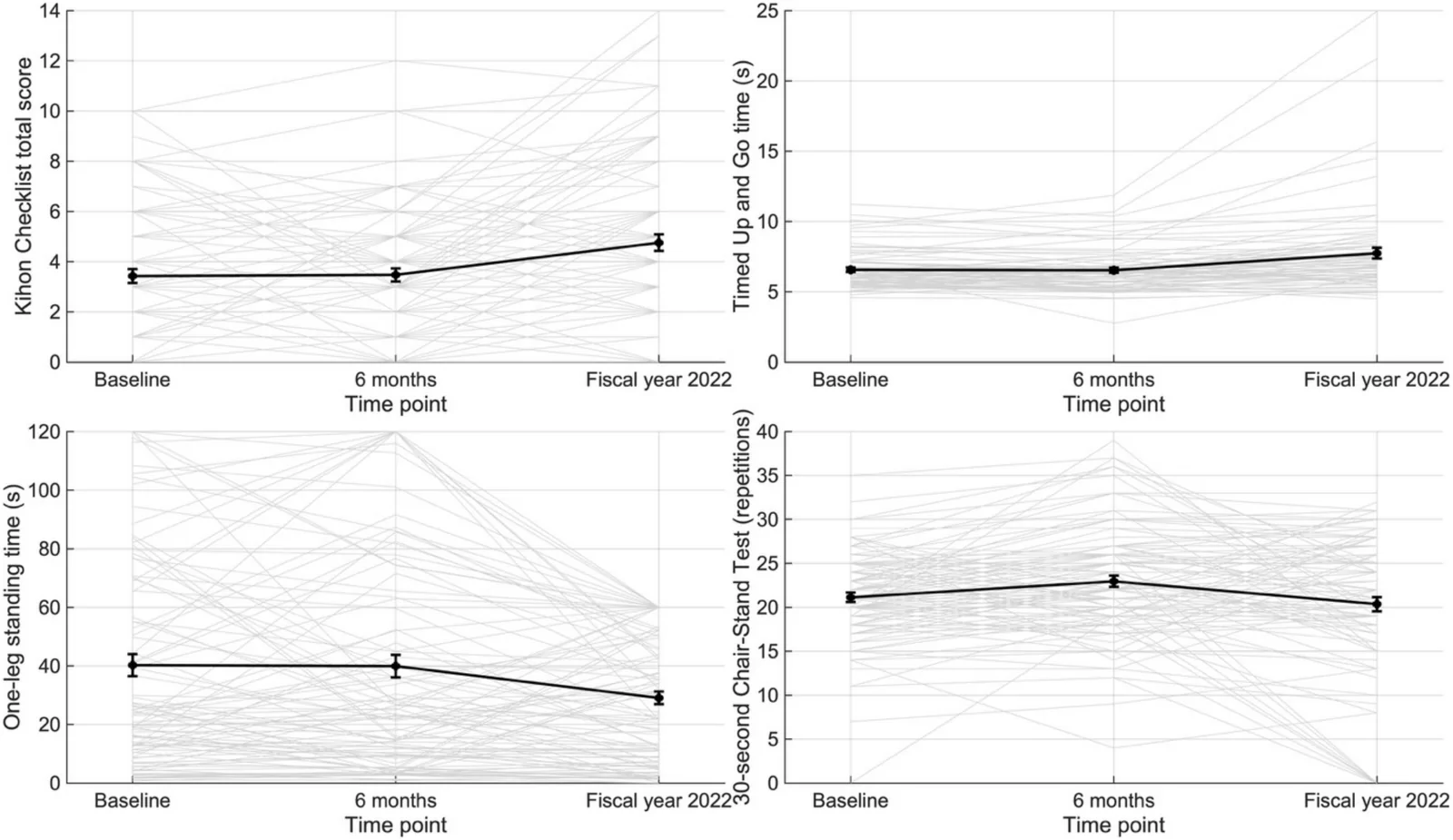
Individual trajectories of frailty and physical function among adults participating in community gathering places in Koshigaya City, Japan, from baseline through the fiscal year 2022 municipal evaluation, conducted between August 2022 and November 2023. The gray lines indicate individual trajectories, whereas the black lines indicate mean values with standard errors. Assessments were conducted at baseline between September 2016 and October 2019, approximately 6 months later between March 2017 and October 2020, and at long-term municipal evaluation between August 2022 and November 2023. Kihon Checklist total score and Timed Up and Go test time increased, whereas one-leg standing time decreased by the long-term evaluation. The 30 s Chair-Stand Test showed initial improvement at approximately 6 months followed by decline. Lower values indicate better status for the Kihon Checklist total score and Timed Up and Go test, whereas higher values indicate better performance for one-leg standing time and the 30 s Chair-Stand Test.

### 3.2 Trajectory patterns

Trajectory distributions differed by outcome (Table 3; Supplementary file 1). CS-30 showed the highest LongGood (51.00%) and ImproveMaintain (38.00%) rates, whereas TUG showed the lowest LongGood rate (27.03%) and frequent ImproveRebound (37.84%) and ConsistentDecline (28.38%) patterns. OLS and KCL had LongGood rates of 33.98% and 38.95%, respectively.

**Table 3:** Distribution of direction-based frailty and physical-function trajectories among adults participating in community gathering places in Koshigaya City, Japan, from baseline through the fiscal year 2022 municipal evaluation, conducted between August 2022 and November 2023.

Outcome	n	Improve Maintain, n (%)	Improve Rebound, n (%)	Consistent Decline, n (%)	Delayed Improve, n (%)	Stable OrOther, n (%)	Long Good, n (%)
Kihon Checklist total score	95	17 (17.89)	10 (10.53)	6 (6.32)	7 (7.37)	55 (57.89)	37 (38.95)
Timed Up and Go Test	74	15 (20.27)	28 (37.84)	21 (28.38)	4 (5.41)	6 (8.11)	20 (27.03)
One-leg standing time	103	24 (23.30)	24 (23.30)	26 (25.24)	11 (10.68)	18 (17.48)	35 (33.98)
30 s Chair-Stand Test	100	38 (38.00)	26 (26.00)	9 (9.00)	13 (13.00)	14 (14.00)	51 (51.00)

LongGood indicates maintenance of, or improvement beyond, baseline status at the FY2022 evaluation. ImproveMaintain indicates initial improvement at 6 months with maintenance of a favorable status at the FY2022 evaluation. Percentages are calculated using outcome-specific complete-case samples. The five direction-based patterns are mutually exclusive. LongGood was analyzed separately as a binary classification for the exploratory discrimination analyses and was not one of the five mutually exclusive trajectory patterns.

Results were broadly similar using the 0.20-SD threshold (Supplementary file 2). Restriction to participants aged ≥65 years yielded similar trajectory distributions, with LongGood rates of 39.33% for KCL (*n* = 89), 26.76% for TUG (*n* = 71), 34.38% for OLS (*n* = 96), and 48.94% for CS-30 (*n* = 94). KCL domain analyses showed greater multidomain worsening among non-LongGood trajectories, whereas LongGood trajectories generally showed improvement or maintenance (Supplementary files 3 and 4).

### 3.3 Exploratory discrimination analysis

Table 4 summarizes the exploratory discrimination analyses. In the apparent internal analyses, the DomainModel showed numerically higher apparent AUCs than the TotalModel for some outcomes. However, these differences were not robust after optimism correction. Several DomainModel AUCs decreased substantially after optimism correction, particularly for TUG outcomes. A graphical summary of apparent and optimism-corrected AUCs is provided in Supplementary file 5, and ROC curves for both LongGood and ImproveMaintain classifications using the DomainModel are provided in Supplementary file 6.

**Table 4:** Exploratory discrimination of favorable long-term trajectories among adults participating in community gathering places in Koshigaya City, Japan, from baseline through the fiscal year 2022 municipal evaluation, conducted between August 2022 and November 2023.

Outcome	Target	Model	n	Events	Predictors	Apparent Area Under the Curve (95% Confidence Interval)	Optimism -corrected Area Under the Curve
Kihon Checklist total score	Long Good	Total Model	87	35	7	0.78 (0.66, 0.88)	0.71
Kihon Checklist total score	Long Good	Domain Model	87	35	13	0.83 (0.74, 0.92)	0.72
Kihon Checklist total score	Improve Maintain	Total Model	87	15	7	0.87 (0.78, 0.95)	0.79
Kihon Checklist total score	Improve Maintain	Domain Model	87	15	13	0.89 (0.78, 0.97)	0.75
Timed Up and Go test	Long Good	Total Model	67	16	7	0.63 (0.48, 0.79)	0.47
Timed Up and Go test	Long Good	Domain Model	67	16	13	0.77 (0.62, 0.90)	0.56
Timed Up and Go test	Improve Maintain	Total Model	67	11	7	0.67 (0.46, 0.86)	0.50
Timed Up and Go test	Improve Maintain	Domain Model	67	11	13	0.88 (0.76, 0.97)	0.71
One-leg standing time	Long Good	Total Model	86	29	7	0.87 (0.80, 0.94)	0.82
One-leg standing time	Long Good	Domain Model	86	29	13	0.90 (0.83, 0.96)	0.81
One-leg standing time	Improve Maintain	Total Model	86	20	7	0.88 (0.80, 0.94)	0.81
One-leg standing time	Improve Maintain	Domain Model	86	20	13	0.89 (0.81, 0.95)	0.76
30 s Chair-Stand Test	Long Good	Total Model	87	43	7	0.79 (0.70, 0.88)	0.73
30 s Chair-Stand Test	Long Good	Domain Model	87	43	13	0.85 (0.77, 0.92)	0.75
30 s Chair-Stand Test	Improve Maintain	Total Model	87	31	7	0.77 (0.67, 0.87)	0.70
30 s Chair-Stand Test	Improve Maintain	Domain Model	87	31	13	0.81 (0.71, 0.89)	0.68

AUC confidence intervals were estimated using 1000 bootstrap resamples. Optimism-corrected AUCs were obtained using bootstrap internal validation. The TotalModel included age, sex, baseline TUG, baseline OLS, baseline CS-30, baseline LSA total score, and baseline KCL total score. The DomainModel replaced the KCL total score with the seven KCL domain scores. Several models had fewer than five events per predictor; therefore, these results should be interpreted as exploratory internal estimates rather than validated prediction performance.

Although the DomainModel showed numerically higher apparent AUCs for some outcomes, attenuation after optimism correction indicated substantial uncertainty and possible overfitting. Therefore, these results should be interpreted as exploratory internal discrimination analyses rather than development of a clinically usable prediction model.

## 4 Discussion

This exploratory longitudinal study identified heterogeneous, outcome-dependent trajectories of frailty and physical function among older adults participating in a municipal Kayoinoba program. CS-30 showed the most favorable trajectory distribution, whereas TUG showed more frequent rebound or decline patterns. These TUG findings should be interpreted in the context of favorable baseline performance and limited room for improvement rather than as evidence of marked deterioration. Because no non-participant comparison group was available, the observed patterns should be interpreted as descriptive trajectories among continuing participants, not as evidence of Kayoinoba effectiveness.

Our findings are consistent with previous studies showing that frailty transitions and physical performance trajectories in older adults are dynamic and rarely uniform (Hoekstra et al., 2020; Imai et al., 2025; Ohashi et al., 2021; Tange et al., 2022; Taniguchi et al., 2016). Although social participation and community gatherings have been associated with favorable functional outcomes (Abe et al., 2023; Hayashi et al., 2021a; Mori et al., 2025; Tajika et al., 2022; Uemura et al., 2025), the present study highlights the diversity of long-term trajectories that average group-level changes can mask. The coexistence of favorable CS-30 patterns and less favorable TUG or KCL patterns suggests that outcome-specific monitoring is needed in real-world community programs.

The relatively favorable CS-30 trajectory profile is clinically noteworthy. The mean baseline CS-30 score in the present sample was 21.14 repetitions, which was higher than the reference value of 17.26 repetitions (95% CI, 15.98, 18.55) reported in a meta-analysis of healthy older Japanese adults (Nakazono et al., 2014). This comparison suggests relatively favorable lower-extremity functional performance at baseline in the present sample. Because CS-30 reflects repeated sit-to-stand ability and lower-limb functional strength (Jones et al., 1999), these findings may be consistent with the exercise-focused nature of Kayoinoba; however, causal inferences cannot be made (Hayashi et al., 2021a, Hayashi et al., 2021b; Mori et al., 2025; Tajika et al., 2022). In contrast, baseline TUG performance was already favorable, suggesting limited room for further improvement. Because TUG reflects multiple mobility components, including standing, walking, turning, and sitting (Podsiadlo and Richardson, 1991), continued monitoring may help identify individuals requiring supplementary support. Sensitivity analyses attenuated some unfavorable classifications, especially for TUG and OLS, supporting interpretation of the categories as descriptive patterns rather than clinically validated change categories.

KCL is clinically relevant because it captures multidimensional frailty-related vulnerability across physical, nutritional, oral, cognitive, depressive, and social domains (Fukutomi et al., 2015; Satake et al., 2016; Sewo Sampaio et al., 2016). Although CS-30 showed relatively favorable trajectory patterns, KCL total score worsened over time, suggesting that physical performance measures alone may not fully capture long-term frailty-related changes (Hirose et al., 2022; Imai et al., 2025; Imai et al., 2023; Ohashi et al., 2021; Yang et al., 2024). Supplementary analyses further supported this interpretation, with unfavorable KCL trajectories associated with worsening across multiple domains and favorable trajectories generally showing improvement or maintenance. Notably, improvement or maintenance of depressive mood, ADL, and physical function among participants with LongGood KCL trajectories suggests that favorable long-term frailty status reflects broader preservation of psychological, functional, and physical domains.

Exploratory discrimination analyses suggested that baseline multidimensional KCL information may contain some discriminatory information beyond the KCL total score. However, the apparent advantage of the DomainModel was attenuated after optimism correction, particularly in models with limited event numbers. Therefore, these findings should be viewed as exploratory signals for future validation rather than evidence of a clinically usable prediction model.

From a public health perspective, repeated multidimensional assessments, including the KCL, may help identify participants who require targeted support beyond group-based activities (Fukutomi et al., 2015; Satake et al., 2016). However, this study describes heterogeneity in long-term trajectories among older adults who continued participation and completed repeated assessments rather than estimating Kayoinoba effectiveness. Future studies with comparison groups, harmonized longitudinal samples, and external validation are needed to determine whether similar patterns are observed in other settings and whether any components of community participation are causally related to them.

A strength of this study was the use of real-world municipal data collected under routine program conditions, together with repeated assessment of multidimensional frailty and physical function. Several limitations should be noted. First, without a non-participant control group, observed trajectories cannot be attributed to Kayoinoba rather than natural aging. Second, participants were volunteers from a single municipality and may have had greater health awareness or functional capacity than the general population, limiting generalizability. Although a small proportion of participants were younger than 65 years, age-restricted sensitivity analyses yielded similar trajectory distributions, suggesting that their inclusion did not materially affect the overall interpretation. Third, outcome-specific complete-case analyses may have introduced selection and attrition bias, potentially overrepresenting favorable trajectories. Fourth, trajectories were based on only three assessments and prespecified rules; the 0.20-SD sensitivity threshold was distribution-based rather than clinically validated. Fifth, longitudinal Life-Space Assessment data were unavailable, and KCL domain analyses were descriptive. Finally, discrimination models used the same data for development and evaluation, had limited event numbers, and included baseline measures related to baseline-referenced outcomes. Consequently, apparent discrimination may partly reflect overfitting, mathematical coupling, regression to the mean, or differences in room for improvement and requires external validation.

## 5 Conclusions

Frailty and physical function showed heterogeneous, outcome-dependent trajectories among Kayoinoba participants. CS-30 showed relatively favorable patterns, whereas TUG more often showed rebound or decline, partly reflecting favorable baseline performance. KCL may complement physical measures by capturing multidimensional frailty-related change. Exploratory discrimination findings require confirmation in larger cohorts with comparison groups and external validation.

## Data statement

The datasets used and/or analyzed during the current study are available from the corresponding author on reasonable request.

## CRediT authorship contribution statement

**Keisuke Kubota:** Writing – original draft, Methodology, Formal analysis, Conceptualization. **Takaya Abe:** Investigation, Data curation. **Takashi Nasu:** Investigation, Data curation. **Yu Shimano:** Investigation, Data curation. **Hiroo Furusawa:** Investigation, Data curation. **Toyohiro Hamaguchi:** Writing – review & editing, Methodology. **Yoshinori Kitabatake:** Writing – review & editing, Methodology. **Yayoi Amakusa:** Writing – review & editing, Methodology.

## Consent for publication

Not applicable.

## Ethics approval and consent to participate

The study was conducted in accordance with the Declaration of Helsinki and was approved by the Ethics Committee of Saitama Prefectural University (approval number: 25101). Written informed consent was obtained from all participants.

## Ethics declaration

### Informed consent and patient details

Written informed consent to take part in the study and to publish the article has been obtained from all participants or their legal representatives. The privacy rights of participants have been observed.

### Studies in human

This study was performed in compliance with relevant laws, regulatory frameworks and guidelines where the research took place. This study was approved by the Saitama Prefectural University. (Approval No. 25101)

## Funding

This work was supported by the Research and Development Center Project, Saitama Prefectural University. The funder had no role in the study design, data collection, analysis, interpretation of data, decision to publish, or preparation of the manuscript.

## Declaration of competing interest

The authors declare that they have no known competing financial interests or personal relationships that could have appeared to influence the work reported in this paper.

## Data Availability

Data will be made available on request.

## Appendix A Supplementary data

Supplementary material 1 Supplementary material includes trajectory pattern distributions, sensitivity analyses, domain-level Kihon Checklist analyses, and exploratory discrimination analyses.
